# Why reporting requires more than findings

**DOI:** 10.1113/EP093705

**Published:** 2026-06-19

**Authors:** Robin Christensen, Tobias Haugegaard, Janus C. Jakobsen

**Affiliations:** ^1^ Section for Biostatistics and Evidence‐Based Research the Parker Institute, Bispebjerg and Frederiksberg Hospital Copenhagen Denmark; ^2^ Department of Clinical Research, Research Unit of Rheumatology University of Southern Denmark, Odense University Hospital Odense Denmark; ^3^ Department of Clinical Research, Cochrane Denmark & Centre for Evidence‐Based Medicine Odense (CEBMO) University of Southern Denmark Odense Denmark; ^4^ Copenhagen Trial Unit, Centre for Clinical Intervention Research, The Capital Region Copenhagen University Hospital – Rigshospitalet Copenhagen Denmark; ^5^ Department of Regional Health Research, The Faculty of Health Sciences University of Southern Denmark Odense Denmark

**Keywords:** misconduct, prespecified analyses, reporting, research, serendipity

## 1 EXPLORATORY SCIENCE, STRUCTURE AND TRANSPARENCY

In what follows, experimental physiology is used in its disciplinary sense – mechanistic biomedical science – not as a reference to the journal *Experimental Physiology*, and the views presented here are the authors’ own and do not constitute official journal policy (although the journal's senior editors originally initiated the discussion through a solicited editorial).

Recent discussions on prespecified analysis plans in experimental physiology have highlighted concerns among basic scientists that greater emphasis on pre‐specification, structured analyses and predefined reporting frameworks may constrain exploratory research and inhibit discovery (Christensen et al., 2025; Richter et al., 2026). Central to this critique is the perception that imposing structure too early could limit flexible interrogation of complex biological data, restrict pursuit of unexpected observations and replace open‐ended exploration with rigid analytic pathways. In this view, pre‐specification is perceived less as a transparency tool than as a potential barrier to discovery. However, substantial evidence indicates that the principal challenge in basic and biomedical science is not exploratory inquiry, but the loss of research value arising from poorly aligned research questions, inefficient study designs, flexible and selective analyses, and incomplete or biased reporting (Chalmers & Glasziou, 2009). Much research proceeds without systematic consideration of prior evidence, resulting in redundancy, underpowered studies, and exaggerated or false‐positive findings that fail to replicate or translate (Chalmers & Glasziou, 2009). When results are reported prematurely or without clear documentation of analytical intent and uncertainty, their scientific value is further diminished.

## 2 EXPLORATION REQUIRES TRANSPARENCY, NOT STATISTICAL ALIBIS

The central challenge, therefore, is not whether exploratory analyses should be permitted, but how early findings are documented, interpreted and communicated. As emphasized by Macleod and colleagues in the 2014 *Lancet* series on reducing waste and increasing value in biomedical research, exploratory research is essential to scientific progress; however, high analytical flexibility combined with weak safeguards against selective reporting increases vulnerability to fragile or misleading findings (Macleod et al., 2014). When applied proportionately, pre‐specification should be viewed not as a constraint on discovery, but to preserve scientific value, protect intellectual priority, and support cumulative knowledge through subsequent confirmation rather than premature overinterpretation.

A related concern is the widespread reliance on null hypothesis significance testing and associated adjustments to police repeated analyses in the absence of full transparency. Statistical tests and associated *P*‐values are not designed to diagnose selective analysis or selective reporting when the full scope of conducted analyses is unknown. Methods intended to address multiplicity, including Bonferroni or related adjustments, presuppose an explicit definition of the family of tests and transparent reporting of all comparisons performed. When analyses are conducted iteratively but only a subset is reported, the effective number of tests cannot be determined, rendering both unadjusted and adjusted *P*‐values difficult to interpret. In such settings, the risk of false‐positive findings becomes unquantifiable rather than merely inflated. Concerns about statistical significance cannot be resolved through *post hoc* threshold manipulation alone, but require transparency about analytical intent, scope and reporting practices. Furthermore, valuable experiments are generalizable beyond the specific study context. When the problem of multiplicity arises, any findings reflect idiosyncratic tendencies in the dataset and are therefore not generalizable. This issue is a potential problem in all forms of research. Importantly, the interpretation of findings should reflect the study's inferential context: exploratory analyses are valuable for hypothesis generation but should not be interpreted as providing confirmatory evidence. Stated plainly, studies not conducted as clinical trials should not be analysed and interpreted as if they were. Basic science research settings may therefore require alternative statistical approaches that do not rely on Fisherian null hypothesis significance testing, which is vulnerable when key inferential assumptions are not met.

## 3 WHEN DOES MISCONDUCT BECOME FRAUD?

Selective omission of analyses from the final report raises concerns that extend beyond statistical interpretation and into the domain of research integrity. When investigators conduct multiple analyses but selectively report only those yielding favourable or statistically significant results, the published record no longer provides an accurate account of the research performed. Under widely accepted standards, including those articulated by the International Committee of Medical Journal Editors (ICMJE), such selective suppression of data or analyses may constitute falsification, defined as misrepresentation of the research record through omission rather than fabrication. The concern is not the conduct of exploratory analyses per se, but the failure to transparently disclose their scope, selection and role in the reported findings. When selective omission materially alters interpretation, such behaviour may meet accepted definitions of falsification and, if intentional, scientific fraud.

Pre‐specification and transparent reporting do not prohibit exploration; rather, they function as safeguards that support accountability by making analytical decisions open to scrutiny. Statistical inference cannot compensate for undisclosed analytical flexibility or selective reporting, and concerns about false‐positive findings cannot be resolved by *post hoc* manipulation of significance thresholds alone. Transparency about what was planned, what was explored and what was ultimately reported is therefore a prerequisite for meaningful interpretation of statistical results (Christensen et al., 2025).

## 4 SEPARATING DISCOVERY FROM DISCLOSURE

An instructive analogy can be drawn from industry‐sponsored research, where early signals are typically not disclosed until appropriate documentation and protection, such as patent filings, are in place. This practice is not intended to constrain discovery, but to ensure that emerging findings are interpretable, attributable and reproducible once they enter the public domain. A substantial proportion of biomedical research fails to deliver cumulative value, not because discoveries lack importance, but because they are lost through avoidable weaknesses in design, analysis and reporting (Chalmers & Glasziou, 2009). These challenges are not confined to clinical trials but apply equally to basic research, where premature dissemination of uncontextualized findings can yield results that are difficult to replicate or confirm.

From this perspective, separating discovery from disclosure may be advantageous rather than restrictive; the scientific literature does not necessarily benefit from the premature publication of preliminary findings. Allowing time to secure funding, conduct prospective confirmation and document analytical intent can protect early findings from premature overinterpretation and loss of intellectual priority.

## 5 AN URGENT NEED TO IMPROVE REPORTING IN EXPERIMENTAL PHYSIOLOGY

If experimental physiology is to generate findings that are interpretable, reproducible and cumulatively valuable, reporting practices must extend beyond selective presentation of results toward transparent documentation of how those results were obtained. Insufficient reporting of design choices, analytical scope and decision‐making processes remains a major threat to the reliability of published findings (Sullivan et al., 2016). Readers must be able to determine how many outcomes, comparisons and statistical tests were conducted before the final tables and figures were assembled. Without this information, a fair interpretation of multiplicity and false‐positive risk is impossible.

## 6 DISCOVERY THRIVES ON TRANSPARENCY

Improving reporting in experimental physiology does not require restricting exploratory research or importing rigid clinical‐trial formalisms into basic science (Richter et al., 2026). Rather, it invites a shared effort to make the work more interpretable by distinguishing planned from exploratory analyses, documenting analytical scope, and being transparent about how results were selected for presentation (Christensen et al., 2025). Such openness supports reproducibility and strengthens confidence in experimental physiology as a collective enterprise. Improved reporting should therefore be understood not as an administrative burden imposed by one community on another, but as a collaborative commitment to stewardship of discovery and preservation of scientific value.

## AUTHOR CONTRIBUTIONS

All authors have read and approved the final version of this manuscript and agree to be accountable for all aspects of the work in ensuring that questions related to the accuracy or integrity of any part of the work are appropriately investigated and resolved. All persons designated as authors qualify for authorship, and all those who qualify for authorship are listed.

## CONFLICT OF INTEREST

The authors declare no conflicts of interest.

## FUNDING INFORMATION

'R.C. and T.H. is partially funded by the Oak Foundation (USA); OFIL‐24‐074. No specific funding was received for this work.'
